# Mental health literacy and quality of life in Iran: a cross-sectional study

**DOI:** 10.1186/s12888-021-03507-5

**Published:** 2021-10-12

**Authors:** Alireza Jafari, Mahbobeh Nejatian, Vahideh Momeniyan, Fatemeh Ramezani Barsalani, Hadi Tehrani

**Affiliations:** 1 Department of Health Education and Health Promotion, School of Health, Social Development and Health Promotion Research Center, Gonabad University of Medical Sciences, Gonabad, Iran; 2 Social Determinants of Health Research Center, Gonabad University of Medical Sciences, Gonabad, Iran; 3 Department of Psychology, Ferdowsi University of Mashhad, Mashhad, Iran; 4 Student Research Committee, Mashhad University of Medical Sciences, Mashhad, Iran; 5 Department of Health Education and Health Promotion, Social Determinants of Health Research Center, Mashhad University of Medical Sciences, Mashhad, Iran

**Keywords:** Mental health; MHLS, SF-12, Mental health literacy, Quality of life

## Abstract

**Background:**

Mental health is one of the effective factors in the quality of life of people. The aim of the present study was to determine the status of mental health literacy (MHL) and its relationship with the quality of life across the Iranian general population.

**Methods:**

In this cross-sectional study, a multi-stage sampling method was used to survey 1070 participants from the city of Gonabad (Iran). The data collection tools were demographics section, mental health literacy scale (MHLS), and quality of life (SF-12) questionnaires. The data was analyzed by SPSS software version 24 using Independent sample t- test, One- way ANOVA, Pearson correlation, and logistic regression.

**Results:**

The mean and standard deviation of the total scores of MHL and quality of life were 113.54 (10.34) and 35.26 (6.42), respectively. The results revealed that there was a significant positive correlation between MHL and the quality of life (*p* < 0.001). In this study, there was a significant relationship between variables of sex, level of education, plus received information about mental illness and MHL (*p* < 0.001). The quality of life was higher in participants whose family members did not have a mental illness, had a high-income level, and received information about mental illness (*p* < 0.001). Logistic regression indicated that there was a significant relationship between the ability to recognize mental disorders plus knowledge of where to seek information and obtaining information related to mental health (*p* < 0.001).

**Conclusion:**

Based on the results of this study, there was a correlation between health literacy and quality of life, and more attention should be paid to MHL. Thus, appropriate programs should be designed and implemented to enhance the level of MHL.

## Background

Mental illness is now an important health problem, whose burden is progressively increasing [1]. The WHO survey of mental disorders in 28 countries revealed that mental disorders were widespread in all population countries, and the prevalence of mental disorders was estimated to be between 18.1 and 36.1% [2]. The results of an international study conducted in 2018 indicated that 35.3% of college students suffer from mental illness throughout their lives. The rates of mental illness in Australia was 48.3, 22.4% in Belgium, 41.1% in Germany, 27% in Mexico, 39.1% in Northern Ireland, 36.1% in South Africa, 39.8% in Spain, and 28.7% in the United States of America [3]. The results of a national study in Iran indicated that about 23% of people suffer from at least one mental disorder [4].

Due to the economic, emotional, suffering and limitations they cause, mental disorders make it difficult for people to perform their social and professional activities [5, 6]. Mental health literacy (MHL) is one of the most important strategies to reduce the burden of mental health disorders [7, 8]. MHL refers to “knowledge and beliefs about mental disorders which aid their recognition, management or prevention” [9]. Increasing the level of MHL facilitates prevention, early detection of disease, ability to intervene, and eventually decrease mental disorders in the community [7, 8]. The results of various studies in China, Vietnam, Nigeria, and Iran have shown that most people have low levels of MHL and only a small percentage have adequate levels of such literacy [1, 10–12].

Health literacy is one of the predictive indicators of quality of life. Improving the level of health literacy helps to improve the quality of life of individuals [13–15]. The results of a meta-analysis study showed that there was a correlation between health literacy and quality of life [16]. Some results indicated that poor health literacy was related to poor quality of life, and this relationship could be due to reduced access to medical care, increased stress due to the challenges of daily living, poor self-management, and reduced self-efficacy [16–19]. On the other hand, because health literacy was affected by many factors (including factors of individual, social, and cultural); any society has reported different results [20–22].

The quality of life interacts with the dimensions of physical and mental health is influenced by various economic, social, mental, and physical factors [23, 24]. Quality of life is a multi-dimensional concept defined by World Health Organization (WHO) as “individuals’ perception of their position in life in the context of the culture and value systems in which they live and in relation to their goals, expectations, standards, and concerns” [25]. This concept has various dimensions that are influenced by the person’s psychological state, physical health, social relationships, personal beliefs, and relationship with their environment [25]. Although many studies have examined the relationship between general health literacy and quality of life, few studies have investigated the relationship between MHL and quality of life [26–28]. The purpose of this study was to determine the status of MHL and its relationship with the quality of life in the general population of Gonabad city, Iran.

## Methods

### Sample size and data collection

This cross-sectional study was performed by using a multi-stage sampling method to survey 1070 participants from the city of Gonabad (Iran). The data collection period was from October 2020 to January 2021. According to a similar study [29], the sample size was estimated to be 1080 participants (95% confidence level, d/accuracy = 0.02, *p* = 0.90%, sample loss rate 20%(. The samples for this study were selected from health centers.

To select the samples, first all health centers and the number of customers in each center were determined. Each center was considered as a class; from each center according to its population, the samples required for the study were randomly selected. Health centers in Iran are different from medical centers. The services provided by the health centers include preventive services for healthy people. The researcher then referred to health centers, and participants were randomly selected based on the inclusion criteria. Before completing the information, the objectives of the study were stated for the participants. If they agree to participate in the study, the questionnaires were provided to the participants and the information was completed by them in a quiet and secluded place in the health center. Also, the information of people who were illiterate was completed by the questioner. Inclusion criteria were no psychopathy or cognitive problems (based on the information recorded in the person’s health record), age 18 years old, completion of written informed consent, and willingness to participate in the study voluntarily.

### Study tools

Data collection tools included questionnaires of demographic, MHL scale, and quality of life.

#### I. Questionnaire of socio-demographic information

This questionnaire includes questions such as age, sex, education level, job, marital status, history of mental illness in the participants’ family, previous referral of participants to a psychologist/psychiatrist for psychological problems, sources of information about mental health, and etc.

#### II. Mental health literacy scale (MHLS)

The MHLS was designed and evaluated in 2015 by O’Connor [30]. The scale has 35 questions and 6 attributes to measures the level of MHL.

- *Ability of individuals to recognize mental disorders:* This attribute consists of eight items (for example: How likely do you think dysthymia is a disorder). This attribute is measured using a 4-point Likert scale (very unlikely, unlikely, likely, very likely) with a score range of 8 to 32.
- *Attitudes that promote the recognition or appropriate help-seeking behavior:* This attribute consists of sixteen items (for example: If I have a mental disorder, I do not like to tell anyone). This attribute is measured using a 5- point Likert scale [(strongly disagree, disagree, neither agree nor disagree, agree, strongly agree) or (definitely willing, probably willing, neither willing nor unwilling, probably unwilling, definitely unwilling)] with a score range of 16 to 80.
- *Knowledge of* self-treatment: This attribute consists of two items (for example: If someone has difficulty managing their emotions such as, becoming very anxious or depressed, how much do you think improving their sleep quality can be beneficial to them?). This attribute is measured using a 4-point Likert scale (very unhelpful, unhelpful, helpful, very helpful) with a score range of 2 to 8.
- *Knowledge of the professional help available*: This attribute consists of three items (for example: In your opinion, it is likely that that Cognitive Behavior Therapy is a therapy based on the challenging negative thoughts and increasing helpful behaviors). This attribute is measured using a 4-point Likert scale (very unlikely, unlikely, likely, very likely) with a score range of 3 to 12.
- *Knowledge of where to seek information*: This attribute consists of four items (for example: I’m sure I can use computers and telephones to seek information about mental disorders). This attribute is measured using a 5-point Likert scale (strongly disagree, disagree, neither agree nor disagree, agree, strongly agree) with a score range of 4 to 20.
- *Knowledge of risk factors and causes:* This attribute consists of two items (for example: In general, to what extent do you think men in Iran may be more experience anxiety disorders more than women). This attribute is measured using a 4-point Likert scale (very unlikely, unlikely, likely, very likely) with a score range of 2 to 8.

A high score for each attribute indicates a higher literacy rate for each attribute. Also, the total score of MHLS is obtained from the sum of the scores of all attributes. The lowest score is 35 while the highest is 160. The higher scores reveal more favorable MHL status. In the O’Connor study, the internal consistency of this tool using Cronbach’s alpha was reported as 0.873 [30]. The validity and reliability of this questionnaire were evaluated by Noroozi et al. in Iran. The Cronbach’s alpha and content validity ratio (CVR) for the questionnaire were 0.72 and 0.90, respectively [31]. The Cronbach’s alpha coefficient ranging from 0.70 to 0.95 is acceptable [32, 33].

#### III. Quality of life questionnaire (SF-12)

The questionnaire is a shortened form of the quality of life questionnaire, designed by Ware and Keller, and widely used in various studies [34]. This questionnaire has 8 sub-scales and examines the quality of life in terms of physical functioning (PF: 2 items, a 3-point Likert scale from “Yes, limited a lot” to “No, not limited at all”), role limitations due to physical problems (RP: 2 items, two-choice response scale, “Yes” or “No”), body pain (BP: 1 item, a 5-point Likert scale from “Not at all” to “Extremely”), general health (GH: 1 item, a 5-point Likert scale from “Excellent” to “Poor”), vitality (VT: 1 item, a 6-point Likert scale from “All of the time” to “None of the time”), social functioning (SF: 1 item, a 5-point Likert scale from “All of the time” to “None of the time”), role limitations due to emotional problems (RE: 2 items, Two-choice response scale, “Yes” or “No”) and perceived mental health (MH: 2 items, a 6-point Likert scale from “All of the time” to “None of the time”).

These 12 items are divided in two subscales of Physical Health with 6 items (RF, RP, BP, GH), and Mental Health with 6 items (SF, RE, VT, MH). The minimum and maximum scores on the Physical Health subscale range between 6 and 20. Also, the minimum and maximum scores on the Mental Health subscale range from 6 to 27. The high scores in each subscale of quality of life indicate favorable status. Also, the total score of quality of life is obtained from the sum of the scores of two subscales (Physical Health and Mental Health). The minimum and maximum scores are between 12 and 47, with high score indicating more favorable quality of life status.

The psychometric properties of this 12-item questionnaire has been approved by Montazeri et al. in Iranian society [35]. The validity and reliability of this questionnaire have been previously confirmed by Montazeri et al. and Cronbach’s alpha for Physical Health subscale and Mental Health subscale were 0.73 and 0.72, respectively [35]. Based the results, the Cronbach’s alpha coefficient ranging from 0.70 to 0.95 is acceptable [32, 33].

### Data analysis

The data was analyzed using SPSS software version 24. Categorical variables included sex, age group, marital status, education level, residence, job, history of mental illness in family, refer to a psychologist/psychiatrist for psychological problems, refer your family members to a psychologist/psychiatrist for psychological problems, income level, and get information related to mental illness. Continuous variables included age, quality of life, and MHL. In the present study, data normality was performed using Kolmogorov Smirnov test and parametric tests (Independent samples t-test, One-way ANOVA, and Pearson correlation) were used according to the normalization of data. Independent samples t-test was used to compares the mean scores between two unrelated groups on the same continuous, dependent variable. One-way ANOVA test was used to compare the mean scores between two or more unrelated groups on the same continuous, dependent variable.

In this study, univariate and multivariate logistic regression were used to investigate the relationship between the dependent variables (yes/no) and MHL attributes. The dependent variables included refer to a psychologist/psychiatrist for psychological problems, get information related to mental illness, and history of mental illness in the family. At first, the each variable was entered in the univariate model and each of them with a significance level of less than 0.2 (*P* < 0.2) was entered into the multivariate model [36]. The significance level of the data analysis was less than 0.05.

## Results

The response rate of this study was 99% and 10 questionnaires were excluded from the study due to incomplete information. Finally, data analysis was performed on 1070 participants. The mean and standard deviation of the participants’ ages was 31.26 and 10.29. The results showed that 44% (*n* = 468) were men and 56% (*n* = 596) were women. Most of the participants were married (68.6%), had college education (67%), urban residents (79.2%), self-employed (53.6%) and middle-income level (66.8%). Only 14.3% of participants (*n* = 151) reported having a family history of mental illness. Also, only 15.6% (*n* = 164) of participants reported that they referred to a psychologist/psychiatrist for psychological problems. In this study, 18% (*n* = 185) of participants reported that one of their family members had referred to a psychologist/psychiatrist for psychological problems.

**Fig. 1:**
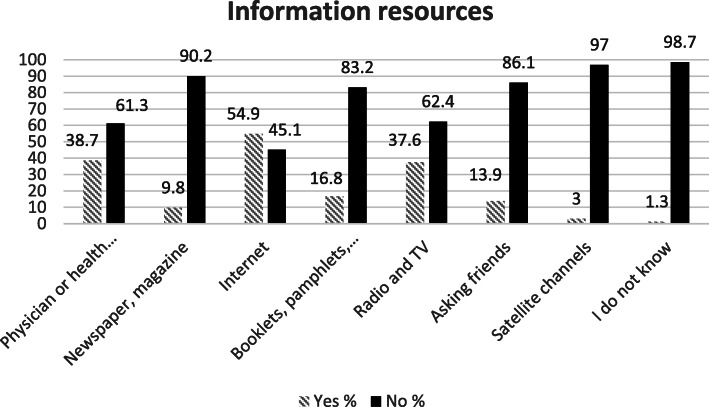
Percentage of information resources related to health

**Fig. 2:**
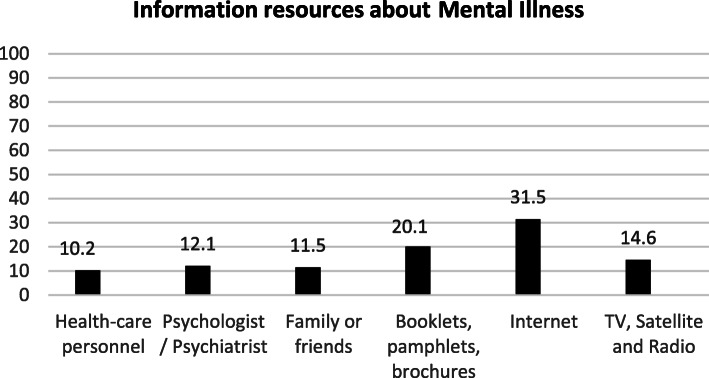
Percentage of information resources related to mental illness

Among the participants, 77.8% (*n* = 826) reported that they have obtained information about mental illnesses (Table 1). Participants obtained health information from the internet (54.9%), physicians/ health care providers (38.7%), and mass media (37.6%) (Fig. 1). Most information about mental illness was obtained from the internet (31.5%), booklets, pamphlets, brochures (20.1%), and mass media (14.6%) (Fig. 2). The Pearson correlation results showed that there was a significant positive correlation between MHL and quality of life (*r* = 0.205, df = 1059, *p* < 0.001). The results also showed a significant positive correlation between MHL and its attributes (*p* < 0.001). Based on the results, there was a significant positive correlation between the quality of life and its subscales (*p* < 0.001) (Table 2).

**Table 1:** Frequency distribution of demographic factors (*N* = 1070)

Variables		n (%)	Quality of Life	***p***-value	MHL	***p***-value
			Mean (SD)		Mean (SD)	
**Sex**	Men	468(44)	35.20(6.52)	0.796*	111.43(10.46)	< 0.001*
	Women	596(56)	35.31(6.35)		115.21(9.81)	
**Age group**	18–26	413(39.1)	35.59(6.11)	0.002**	113.92(10.17)	0.031**
	27–35	291(27.5)	35.95(6.43)		114.37(10.35)	
	36–44	223(21.1)	34.80(6.51)		113.54(10.06)	
	45 and above	130(12.3)	33.5696.67)		111.25(10.4)	
**Marital status**	Marriage	724(68.6)	35.07(6.37)	0.129*	113.49(10.25)	0.625*
	Single	332(31.4)	35.72(6.47)		113.83(10.57)	
**Education level**	Illiteracy	7(0.7)	37.71(7.31)	< 0.001**	105.14(12.01)	< 0.001**
	Elementary	23(2.2)	35.00(6.250		107.65(11.01)	
	High school	310(30.1)	33.55(6.35)		110.79910.07)	
	Academic	691(67)	36.11(6.30)		115.19(10.05)	
**Residence**	Urban	787(79.2)	35.37(6.39)	0.300*	113.90(10.61)	0.186*
	Rural	207(20.8)	35.88(6.36)		112.91(9.27)	
**Job**	Housewife	158(15.4)	34.19(6.55)	0.051**	112(80(9.29)	0.224**
	Employed	317(31)	35.69(6.60)		114.38(11)	
	Self-employed	549(53.6)	35.40(6.25)		113.38(10.23)	
**History of mental illness in your family**	Yes	151(14.3)	32.56(6.40)	< 0.001*	112.58(11.08)	0.220*
	No	905(85.7)	35.73(6.31)		113.70(10.22)	
**Refer to a psychologist / psychiatrist for psychological problems**	Yes	164(15.6)	32.17(6.28)	< 0.001*	113.75(11.30)	0.761*
	No	888(84.4)	35.86(6.26)		113.48(10.19)	
**Refer your family members to a psychologist / psychiatrist for psychological problems**	Yes	185(18)	33.67(6.56)	< 0.001*	114.27(10.42)	0.307*
	No	845(82)	35.72(6.30)		113.00(10.28)	
**Income**	Good	233(23.2)	37.09(6.08)	< 0.001**	114.71(11.98)	0.124**
	Middle	670(66.8)	35.15(6.25)		113.61(9.61)	
	Weak	100(10)	32.75(6.92)		112.30(9.96)	
**Get information related to mental illness**	Yes	826(77.8)	35.53(6.27)	0.012*	114.49(9.91)	< 0.001*
	No	236(22.2)	34.34(6.79)		110.48(11.040	

* Independent Samples t Test, ** One-way ANOVA test, ^ In some items, the information was not completed by the participants and was miss information. For this reason, the sum of some items does not reach 1070.

**Table 2:** Pearson correlation between attributes of mental health literacy and quality of life

Variables		a	b	c	d	e	f	g	h	i
**Attributes of MHLS**	**Ability of individuals to recognize mental disorders (a)**	1								
	**knowledge of where to seek information (b)**	0.332**	1							
	**Knowledge of risk factors and causes (c)**	0.072*	0.091*	1						
	**Knowledge of self-treatment (d)**	0.070*	0.022	−0.061*	1					
	**Knowledge of the professional help available (e)**	0.361**	0.192**	0.131**	−0.004	1				
	**Attitudes that promote recognition or appropriate help seeking behaviour (f)**	0.136**	0.039	0.018	0.078*	0.146**	1			
**Total mental health literacy(g)**		0.590**	0.447**	0.181**	0.167**	0.417**	0.813**	1		
**Quality of Life(h)**		0.049	0.151**	0.084*	0.036	0.123**	0.165**	0.205**	1	
**Subscales of quality of life**	**Physical health (i)**	0.091	0.177**	0.72*	0.011	0.129**	0.199**	0.248**	0.846**	1
	**Mental health (j)**	0.010	0.103**	0.081*	0.047	0.090*	0.111**	0.136**	0.915**	0.559**

*p < 0.005, **p < 0.001

Based on the results of the Independent samples t-test, there was a significant relationship between sex and MHL, where MHL was higher in women than in men (*p* < 0.001). The results of One-way ANOVA test revealed that there was a significant relationship between the educational level and MHL, where MHL was higher in people with an academic education level (*p* < 0.001). Participants whose family members did not have a mental illness, had high education, had a high-income level, and received information about mental illness had a higher quality of life (*p* < 0.001). Also, based on the results of Independent samples t-test, people who received information about mental illness had a significantly higher MHL level (*p* < 0.001) (Table 1).

The mean and standard deviation of the total score of MHL was 113.54 (10.34), attributes of ability to recognize mental disorders was 3.79 (3.55), knowledge of where to seek information was 13.57 (2.78), knowledge of risk factors and causes was 5.34 (1.12), knowledge of self-treatment was 5.16 (0.90), knowledge of professional help available was 8.38 (1.24), and attitudes that promote recognition or appropriate help-seeking behavior was 57.27 (7.54). The mean and SD of the total score of quality of life was 35.26 (6.42), Physical health subscale was 15.52 (3.10), and Mental health subscale was 19.75 (4.09) (Table 3).

**Table 3:** Descriptive characteristic of the mental health literacy and quality of life

Variables		Item	Mean	SD	Range	Cronbach’s alpha
**Attributes of MHLS**	**Ability of individuals to recogniseze mental disorders**	8	23.79	3.55	8–32	0.75
	**knowledge of where to seek information**	4	13.57	2.78	4–20	0.61
	**Knowledge of risk factors and causes**	2	5.34	1.12	2–8	0.61
	**Knowledge of self-treatment**	2	5.16	0.90	2–8	0.60
	**Knowledge of professional help available**	3	8.38	1.24	3–12	0.60
	**Attitudes that promote recognition or appropriate help seeking behaviour**	16	57.27	7.54	16–80	0.82
**Total score of MHL**		35	113.54	10.34	35–160	0.79
**Subscales of quality of life**	**Physical health**	6	15.52	3.10	6–20	0.72
	**Mental health**	6	19.75	4.09	6–27	0.74
**Total score of quality of life**		12	35.26	6.42	12–47	0.83

The results of logistic regression indicated that there was a significant relationship between the ability to recognize mental disorders and previous referral of participants to a psychologist/psychiatrist for psychological problems (*p* < 0.05, OR = 0.931, CI: 0.881–0.984) (Table 4). There was also a significant relationship between the ability to recognize mental disorders (*p* < 0.001, OR = 0.917, CI: 0.875–0.960) and knowledge of where to seek information (*p* < 0.001, OR = 0.906, CI: 0.857–0.958) with getting information related to mental illness (Table 5). The results also indicated that there was a significant relationship between the ability to recognize mental disorders (*p* < 0.05, OR = 0.916, CI: 0.865–0.969) plus knowledge of professional help available (*p* < 0.05, OR = 1.017, CI: 1.046–1.409) and a history of mental illness in the participants’ family (Table 6).

**Table 4:** Binary logistic regression analysis of refer to a psychologist/psychiatrist for psychological problems

Variables	Refer to a psychologist/psychiatrist for psychological problems*					
	Univariate Regression			Multivariate Regression		
	OR	95% C.I	***P***-Value	OR	95% C.I	***P***-Value
**Ability of individuals to recognize mental disorders**	0.945	0.900–0.992	0.023	0.952	0.904–0.986	0.049
**Knowledge of where to seek information**	0.954	0.897–1.014	0.132	0.973	0.911–1.038	0.402
**Knowledge of risk factors and causes**	1.057	0.911–1.227	0.462	–	–	–
**Knowledge of self-treatment**	1.023	0.851–1.229	0.808	–	–	–
**Knowledge of professional help available**	1.064	0.933–1.214	0.356	–	–	–
**Attitudes that promote recognition or appropriate help seeking behaviour**	1.010	0.989–1.032	0.359	–	–	–

*The dichotomous dependent variable (Yes, No)

**Table 5:** Binary logistic regression analysis of get information related to mental illness

Variables	Get information related to mental illness*					
	Univariate Regression			Multivariate Regression		
	OR	95% C.I	***P***-Value	OR	95% C.I	***P***-Value
**Ability of individuals to recognize mental disorders**	0.881	0.846–0.918	< 0.001	0.916	0.875–0.960	< 0.001
**Knowledge of where to seek information**	0.862	0.819–0.908	< 0.001	0.906	0.858–.0.958	0.001
**Knowledge of risk factors and causes**	0.816	0.717–0.930	0.002	0.864	0.756–0.986	0.030
**Knowledge of self-treatment**	1.004	0.856–1.179	0.957	–	–	–
**Knowledge of professional help available**	0.796	0.710–0.893	< 0.001	0.925	0.816–1.050	0.230
**Attitudes that promote recognition or appropriate help seeking behaviour**	0.990	0.971–1.009	0.284	–	–	–

*The dichotomous dependent variable (Yes, No)

**Table 6:** Binary logistic regression analysis of someone with a mental illness in your family

Variables	History of mental illness in the family*					
	Univariate Regression			Multivariate Regression		
	OR	95% C.I	***P***-Value	OR	95% C.I	***P***-Value
**Ability of individuals to recognize mental disorders**	0.958	0.911–1.007	0.095	0.928	0.879–0.980	0.007
**Knowledge of where to seek information**	1.032	0.971–1.096	0.315	–	–	–
**Knowledge of risk factors and causes**	1.036	0.887–1.209	0.657	–	–	–
**Knowledge of self-treatment**	1.070	884–1.296	0.488	–	–	–
**Knowledge of professional help available**	1.153	1.007–1.320	0.039	1.222	1.055–1.416	0.007
**Attitudes that promote recognition or appropriate help seeking behaviour**	1.018	0.996–1.041	0.109	1.017	0.995–1.040	0.126

*The dichotomous dependent variable (Yes, No)

## Discussion

This study aimed to investigate the status of MHL and its relationship with the quality of life of the general population. In this study, the mean and standard deviation of the total score of MHL was 113.54 and 10.34 (out of 160). The mean and standard deviation of the total score of quality of life was 35.26 and 6.42 (out of 47). Also, the results showed that there was a significant positive correlation between MHL and quality of life.

Based on the results obtained in this study, most people did not have a high level of MHL. In other studies conducted by different groups, MHL was not appropriate either. For example, the results of Thai’s study on Vietnamese students indicated that students did not have adequate levels of MHL [1]. The results of a national study conducted by Huang showed that the Chinese population had a low ability to recognize mental illness [10]. In a study by Lam, only 16% of adolescents had a sufficient level of MHL [29]. Al-Yateem’s study on school nurses reported that less than 50% were able to recognize the presented mental disorders and the level of MHL was not optimal [37].

In the present study, most of the information related to mental health and mental disorders was obtained from the Internet, books, and mass media (radio and television), respectively. Li obtained similar results to our study. In Li′s study, the participants who had contact with patients suffering from mental disorders reported that they had obtained information about mental disorders from the mass media [38]. The results of another study showed that people who helped search for mental health received their required information from family and friends rather than mental health professionals [39]. The results of Ghadirian’s study also showed that most people had asked friends and family for help when using mental health services, respectively [40]. People achieve mental health information from various sources that may not be reliable. Psychologists, psychiatrists, and health care providers are the best source of information on mental health, from which people should obtain reliable information [41].

The results of this study showed that there was a significant relationship between sex and MHL, and MHL was higher in women than men. The results of this study were consistent with the results of other studies. The results of various studies showed that women had a higher level of MHL than men [42, 43], had a greater ability to recognize mental disorders [40, 44], and were more likely to seek professional mental health services than men [45]. Also, women tend to know more about mental health and are more inclined to interact with people with mental disorders [46, 47].

In this study, there was a significant relationship between education level and MHL, and MHL was higher in people with academic education. The results of various studies showed that there was a significant relationship between education level and MHL and with increasing education level, MHL level also improves [38, 48]. Also, with an increasing education level, the ability to recognize mental disorders significantly has improved significantly [40, 44].

The results of this study indicated that there was a significant relationship between educational level and quality of life; the quality of life in people with academic education was higher than other people. Similar to the results of this study, a study conducted by Hu showed that with elevation of the educational level, the quality of life in the physical and psychological dimensions increased significantly [49]. The educational level is an important factor in predicting quality of life, with increasing literacy level, quality of life increased significantly [50, 51].

In this study, there was a significant relationship between income level and quality of life, and people with higher income levels also had a higher quality of life. Similar to the results of this study, the results of Ran’s study also showed that income level was one of the important factors in increasing the quality of life and increasing income significantly helps to improve the quality of life [17]. Income level is one of the important factors affecting the quality of life and with increase of income level, the quality of life improves [52].

In the present study, there was a significant positive correlation between MHL and quality of life. In this study, those who received information about mental illness had a significantly higher quality of life. The results of this study were similar with the results of other studies. The results of Ran’s study indicated that there was a significant relationship between health literacy and quality of life, where people with higher levels of health literacy had also a higher quality of life [17]. A study on diabetic patients showed that health literacy is related to the quality of life, and insufficient health literacy can lead to a decline in the quality of life, especially in the field of mental health [53]. Health literacy is one of the important factors in predicting the quality of life, and increasing the level of health literacy significantly enhanced the quality of life in society [14, 50, 51].

Based on the results of this study, there was a significant relationship between referring to a psychologist/psychiatrist for mental problems, and the ability of people to recognize mental disorders, and those who referred had a greater ability to recognize mental disorders. The results of a study among people with depressive symptoms indicated that only 18 to 24% of them were looking for help from specialists [54]. The ability to recognize mental disorders is the first step in seeking information and asking for help, which ultimately leads the person to seek available treatment [40]. Due to the social stigma and embarrassment of mental disorders, many people usually refuse to see a psychologist/psychiatrist or seek professional help [54]. Consulting with a psychologist/psychiatrist could help a person identify risk factors for the disease and ways to prevent the disease.

The results of this study revealed that there was a significant relationship between obtaining information about mental disorders and the ability to recognize mental disorders. Those who obtained more information in this field had a higher ability to recognize mental disorders. The results of this study were consistent with the findings of other studies. The results of a systematic review study by Gulliver indicated that one of the main barriers in not seeking information related to mental disorders was low MHL and inability to recognize mental disorders [54]. The results of the Yu’s study suggested that people with higher knowledge about mental disorders were more likely to seek mental health services [45]. Having higher knowledge in the field of mental health increases the likelihood of a person seeking mental health services [55]. A study finding revealed that many people are unable to recognize specific mental disorders or different types of mental disorders [9]. The results of a study conducted by Jorm et al. on the Australian people showed that people who had a better ability to recognize schizophrenia and depression were more likely to receive a wide range of mental health services, including help from mental health providers, psychotherapy, medications, and psychiatric admissions [56]. Also, there is evidence that the ability to correctly recognize mental disorders is related to using official resources to seek help, and it is more likely to seek information/help from mental health professionals [57].

Based on the results of this study, there was a significant relationship between obtaining information about mental disorders and knowledge of where to seek information, and those who had more information in this field had higher knowledge in searching for information about mental disorders. The results of this study were similar with the results of other studies. The results of a study showed that people had more intentions to seek mental health services, but they had low knowledge about help sources and do not know where to seek help sources [45]. Based on the results of a systematic review study, improve people’s knowledge about mental disorders/mental health and where to seek help and treatment, improving the mental health outcomes and increase the use of mental health services by people [58].

The results of this study demonstrated that there was a significant relationship between having a family history of mental illness and the ability to recognize mental disorders, whereby those who had a family history of mental illness had a higher ability to recognize mental disorders. The results of this study were consistent with the results of other studies. A study reported that people who have contact with patients with mental disorders had a higher ability to recognize mental disorders [44]. The results of various studies have shown that people who have been in contact with the mentally ill have a higher level of MHL than those who have not been in contact [38, 42, 59]. The results of a study revealed that people with a family history were at high risk of mental illness [60]. The risk of depression in people with a history of depression for several generations (multiple generations) in the family increased from 12.6 to 41.4% [60]. Thus, these people must be able to correctly identify mental illnesses to recognize their illness in the early stages and to prevent the progression of the condition.

Based on the results of this study, there was a significant relationship between the history of mental illness in the family and knowledge about professional help available in this field. Participants who reported that one of their family members had a mental disorder had higher levels of knowledge of professional help available related to mental health. Most patients with mental disorder usually live with their family, but most family members have low awareness and literacy about mental health, which is a major challenge for the patient, family, and mental health providers [61]. The higher MHL of family members can enhance their social support for mental illness and help patients seek professional help in this field [62].

### Strengths and limitations

Similar to other studies, this study had some limitations. In this study, information was collected using a questionnaire by self-reporting where there may have been recall bias and reporting bias by participants. This study was cross-sectional and it was not possible to examine the causality between the variables. One of the strengths of this study was the large sample size. Also, in the present study, samples from different age and sex groups participated. It is suggested that in future research, the current status of MHL be investigated across different social groups.

## Conclusion

Based on the results of this study, there was a correlation between health literacy and quality of life, and more attention needs to be paid to MHL. Thus, appropriate plans should be designed and implemented to improve the level of MHL. Due to the inherent limitations and potential biases of this research, it is recommended to be careful when interpreting the results.

## Data Availability

The data sets used and/or analyzed during the current study were available from the corresponding author on reasonable request.

## Abbreviations

MHLS: Mental health literacy scale
MHL: Mental health literacy
SF-12: The 12-item short form health survey
PF: Physical functioning
GH: General health
BP: Body pain
SF: Social functioning
VT: Vitality
MH: Perceived mental health
RE: Role limitations due to emotional problems
RP: Role limitations due to physical problems
